# Laveran et l’éradication du paludisme en Corse

**DOI:** 10.48327/mtsi.v3i1.2023.309

**Published:** 2023-02-06

**Authors:** Pierre Gazin

**Affiliations:** SFMTSI Société francophone de médecine tropicale et santé internationale (ancienne SPE), Hôpital Pitié-Salpêtrière, Pavillon Laveran, 47-83 Boulevard de l'Hôpital, 75651 Paris cedex 13, France; * Actes du Colloque – Centenaire de la mort d'Alphonse Laveran. 24 novembre 2022, Paris / roceedings of the Conference – Centenary of the death of Alphonse Laveran. 24 November 2022, Paris

**Keywords:** Paludisme, Alphonse Laveran, Éradication, Élimination, *Anopheles labranchiae*, DDT, Corse, France, Malaria, Alphonse Laveran, Eradication, Elimination, *Anopheles labranchiae*, DDT, Corsica, France

## Abstract

L'observation de miasmes et de fièvres est attestée dans la région de Biguglia, au sud de Bastia, en 1499, confirmée au cours du XVII^e^ siècle. A partir de 1770, des travaux de drainage sur la côte orientale sont commencés, abandonnés dans la période révolutionnaire, recommencés sous le Second Empire, avec peu de résultats sur l'endémie. En 1875, 80% des habitants de la plaine orientale sont considérés paludéens sur leur aspect. La population rurale est misérable, la mortalité élevée sans que l'on puisse distinguer la part de responsabilité du paludisme parmi les autres fièvres.

En 1899, A. Laveran confirme la présence d'anophèles dans les localités corses où le paludisme sévit. Il impulse la création à Bastia en 1902 de la Ligue corse contre le paludisme et il la préside. Les actions reposent sur la lutte contre les larves par destruction chimique, l'emploi de moustiquaires et sur la « quininisation » préventive massive et gratuite. Une loi d'assainissement de la Corse est votée en 1911. De premiers résultats sont observés, confirmés par Léger et Arlo (1913) [6]. Reprise des actions à partir de 1920 en particulier par les frères Sergent. Une station d'application antipaludique est créée à Bastia en 1925, avec le soutien du laboratoire de parasitologie de la faculté de Médecine de Paris. *Plasmodium falciparum* prédomine, transmis essentiellement par *Anopheles labranchiae* du complexe *maculipennis*, présent jusqu’à une altitude de 500 m.

La libération de la Corse en octobre 1943 permet à l'armée américaine d'installer de nombreux terrains d'aviation, en particulier sur la plaine orientale. Une intense lutte antimoustique de proximité par DDT est alors réalisée et elle marque les esprits. Cependant, le paludisme prospère sur l’île avec une acmé des indices en 1947. A partir de 1948, des campagnes de pulvérisation d'insecticide contre les adultes, de lutte chimique ou par poissons larvivores contre les larves, le traitement des malades dans les dispensaires aboutissent à des résultats très nets dès 1953. Actuellement, la situation en Corse d'anophèlisme sans paludisme est considérée comme étant sous contrôle avec un faible risque de reprise d'une transmission localisée.

## Abstract (en)

The observation of miasmas and fevers was attested in the region of Biguglia, South of Bastia, in 1499, confirmed during the 17^th^ century. Drainage works on the eastern coast were started in 1770, abandoned during the revolutionary period, restarted under the Second Empire, with few results on the endemic. Thus in 1875, 80% of the inhabitants of the eastern plain were considered on their appearance to suffer malaria. The rural population was miserable, the mortality high. However, it was not possible to distinguish the responsibility of malaria among the other fevers.

In 1899 and following years, A. Laveran was in Corsica. He confirmed the presence of Anopheles in the localities where malaria was present. He encouraged the creation in Bastia in 1902 of the Corsican League against Malaria and he chaired it. The actions of this League were based on the fight against the larvae by chemical destruction, on the use of mosquito nets and on massive and free preventive “quininisation”. A sanitation and development law for Corsica was passed in 1911. Initial results were observed, confirmed by Léger and Arlo (1913) [6]. After WW1, the activities started again, in particular by Sergent and Sergent. An antimalarial application station, subsidized by the Rockefeller Foundation, was created in Bastia in 1925, supported by the laboratory of parasitology of the Faculty of Medicine of Paris. *Plasmodium falciparum* was predominant, transmitted essentially by *Anopheles labranchiae* of the *maculipennis* complex, up to an altitude of 500 m. The role of population displacements, linked to pastoral practices, the absence of stables and therefore of zoonotic deviation of anopheles were underlined.

The liberation of Corsica in October 1943 allowed the installation by the American army of numerous airfields on the eastern plain. An intense local mosquito control by DDT was then carried out, impressing the population. However, malaria prospered on the island with an acme of indices in 1947. From 1948, campaigns of spraying insecticide against adults, chemical control of larvae or use of larvivorous fish, treatment of patients in dispensaries led to very good results. Since 1953, malaria transmission is interrupted in Corsica excepted 30 indigenous cases in 1970-71. Currently, the situation in Corsica of an anophelism without malaria is considered to be under control with a low risk of resumption of a localized transmission.

Il est difficile d'affirmer la présence ou l'absence de fièvres palustres dans la Corse romaine ainsi que durant les siècles suivants du fait du manque de documents et de la succession de pouvoirs sur l’île. À partir du xiV siècle, la domination génoise s'affirme progressivement et s'organise. L'observation de miasmes est ainsi attestée dans la région de Biguglia, au sud de Bastia, en 1499. L'existence des fièvres palustres est confirmée au cours des xvii^e^ et xviii^e^ siècles. Changement de pouvoir en 1769 et, à partir de 1770, des travaux de drainage sur la côte orientale sont commencés. Ils sont abandonnés durant la période révolutionnaire, et reprennent sous le Second Empire. Cependant, ils n'ont que peu ou pas de répercussions sur l'endémie palustre. Ainsi en 1875, 80% des habitants de la plaine orientale sont considérés comme paludéens sur leur aspect (« pâleur terreuse, faciès bouffi des habitants de la plaine ») contrastant avec la « bonne mine » des montagnards. La population rurale est misérable, et la mortalité élevée, sans que l'on puisse distinguer la part de responsabilité du paludisme parmi les autres fièvres dont la typhoïde. L'espérance de vie serait de 24 ans à Aleria et de 41 ans à Piedicroce (altitude 650 m) [5, 8]. Les prisonniers du pénitencier agricole de Casabianda ont un taux annuel de mortalité de 10%, en grande partie à cause du paludisme, entraînant sa fermeture en 1886.

## Laveran Et La Corse

Alphonse Laveran effectue plusieurs voyages en Corse entre 1899 et 1903. Il observe l'abondance d’*Anopheles maculipennis* dans les zones insalubres de la côte orientale et sa rareté dans les zones où le paludisme est absent, confortant la théorie anophélienne de la transmission du paludisme. Cette théorie est alors encore contestée malgré les travaux de Ross et de Bignami et Grassi. En 1901, dans un rapport présenté à l'Académie de médecine, A. Laveran demande la création d'une Société pour l'assainissement de la Corse. La Ligue contre le paludisme est créée à Bastia en 1902 par le Dr F. Battesti, et A. Laveran en assure la présidence. Cette société est une structure active et efficace contre le paludisme. Elle réalise des actions concrètes de destruction des gîtes larvaires, d'installation de moustiquaires de fenêtres et portes dans la plaine orientale, de distribution gratuite de quinine en usage préventif (0,25 à 0,4 g/jour), d'enseignement sur la maladie par l'intermédiaire des écoles primaires [7, 10]. Le décès prématuré de Battesti en 1906 entraîne une réduction de ses activités.

Une « Loi d'assainissement de la Corse » est votée fin 1911. Des campagnes en 1912 et 1913 mettent en évidence la présence intense des anophèles dans la plaine orientale ainsi que ponctuellement sur la côte occidentale et dans la plaine centrale. L'importance des petites mares et des gîtes artificiels comme les alentours des puits et fontaines, les bassins pour l'arrosage est soulignée. Les migrations estivales d'une grande partie de la population de la plaine orientale vers la montagne sont stigmatisées, considérées comme responsables de la pauvreté de l’île. L'efficacité de la quininisation préventive est quantifiée [6].

## L'entre-deux-guerres

Les activités contre le paludisme reprennent à partir de 1920. Les conséquences de l'insalubrité sur le développement de l’île sont alors soulignées [1]. La situation n'est pas meilleure que 20 ans auparavant: dans la plaine orientale, indice splénique entre 30 et 50% chez les moins de 16 ans, indices plasmodiques entre 20 et 40%, indices sporozoïtiques entre 0,5 et 1,2%, morbidité importante, faible utilisation de la quinine en prophylaxie systématique malgré sa gratuité, absence de moustiquaires efficaces [9]. *Plasmodium falciparum* prédomine, suivi de *P. vivax* et de quelques cas de *P. malariae.* Des cas de cachexie palustre sont observés. Il n'est cependant pas rapporté de données sur des décès au cours d'accès palustre aigu. Les larves d'anophèles ne sont pas présentes dans les grandes lagunes mais dans leurs canaux d'assèchement mal entretenus, dans les petites mares, dans des gîtes péridomestiques (alentours des fontaines et des puits, bassins de stockage), dans les barques à terre. La transmission s'effectue de juillet à décembre, principalement par *An. maculipennis.* Dans les bons gîtes, on observe de 300 à 500 larves au m^2^. Ces anophèles anthropophiles présentent peu de déviation trophique, sauf dans les porcheries, une situation que la rareté de la stabulation du bétail peut expliquer. Le passage de la saison froide est assuré par quelques femelles fécondées. La prophylaxie par la quinine et l'instruction de la population apparaissent comme les armes les plus réalistes contre ce paludisme intense et focalisé. Une station d'application antipaludique, subventionnée par la Fondation Rockefeller et en lien avec le laboratoire de Parasitologie de la Faculté de médecine de Paris (E. Brumpt), est construite à Bastia en 1925. Elle est à l'origine du Service de lutte antipaludique (1930).

La lutte contre les larves est développée avec l'emploi de « vert de Paris » (acéto-arsénite de cuivre) mélangé à de la « poussière de route ». Les dispersions sont effectuées toutes les deux semaines. Le pétrole ou l'huile de paraffine, voire l'huile d'olive, sont également utilisés. Les poissons larvivores *Gambusia*, introduits en 1926, se révèlent efficaces. Les moustiquaires de fenêtre sont préconisées. L'efficacité de la quininisation de masse et gratuite (0,4 g/jour) est vérifiée (et déjà utilisée à large échelle dans la Double en 1863). La sensibilisation de la population et son accès aux soins sont développés. Les grands travaux d'assainissement se révèlent rarement efficaces, à l'exception du domaine de Casabianda où les travaux ont commencé dès le début du siècle. La mise en culture intensive de la plaine orientale reste un but économique et elle est considérée comme un moyen de réduire les gîtes anophéliens. Toutes ces mesures demandent des moyens humains et financiers importants, la continuité des actions, ce qui n'est pas toujours le cas.

## La Guerre Et L'après-guerre

Le retour de la guerre isole la Corse du reste de la France, entraînant un arrêt des travaux du Service de lutte antipaludique. L'occupation italienne n'est pas particulièrement tendre. La Corse est libérée dès octobre 1943 de ses troupes italiennes et allemandes. L'armée américaine installe de nombreux terrains d'aviation temporaires, particulièrement dans la plaine orientale face à l'Italie et à ses lignes de défense allemandes. C'est l’« USS Corsica », l’île porte-avions. L'armée américaine protège ses hommes de la nuisance des moustiques et du risque de transmission du paludisme par l'emploi massif d'insecticide chimique, une grande nouveauté. Cela impressionne la population et est à l'origine de la légende de « l’éradication du paludisme par les Américains en quelques semaines », récit qui persiste jusqu’à maintenant.

En réalité, le paludisme est toujours présent et il atteint son acmé en 1946-1947 dans une Corse pauvre, aux 3/5 encore rurale et qui se dépeuple (295 000 habitants en 1901, 245 000 en 1954, mortalité infantile 92 pour 1000 en 1947) [4].

À partir de 1947 commencent les activités qui vont aboutir à l'interruption de la transmission en moins de 10 ans [5]. Outre un financement étatique, par la Sécurité sociale et par la Mutualité agricole, elles bénéficient du soutien de la Fondation Rockefeller et du Fonds de secours à l'enfance des Nations unies.

**Figure 1:**
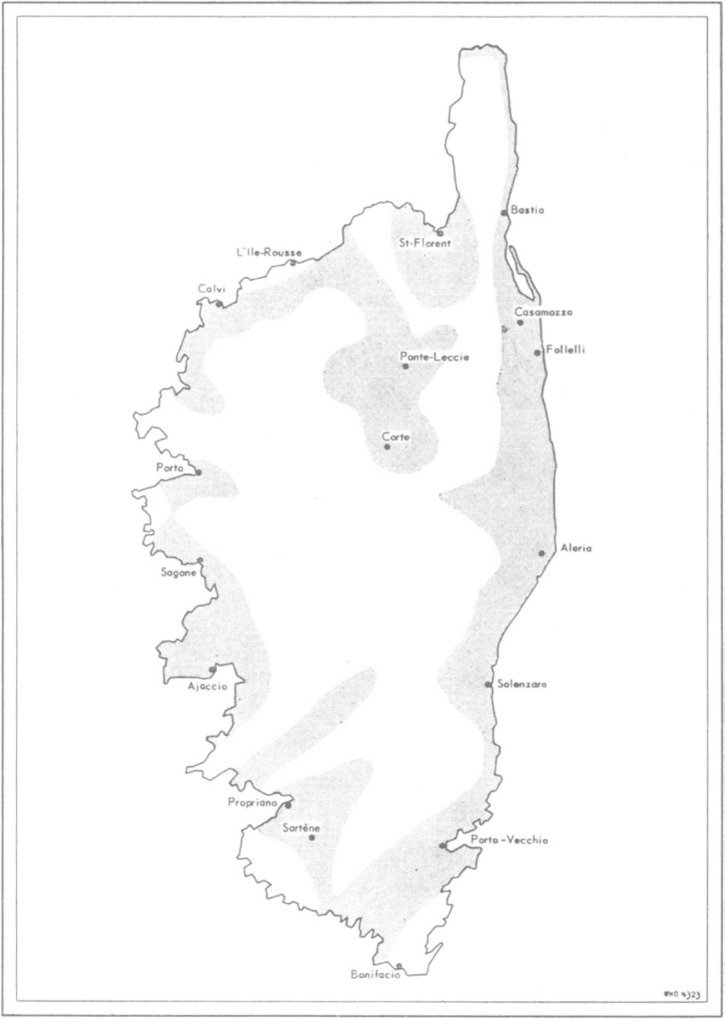
Répartition d'Anopheles labranchiae [5] Anopheles labranchiae distribution in Corsica [5]

**Figure 2:**
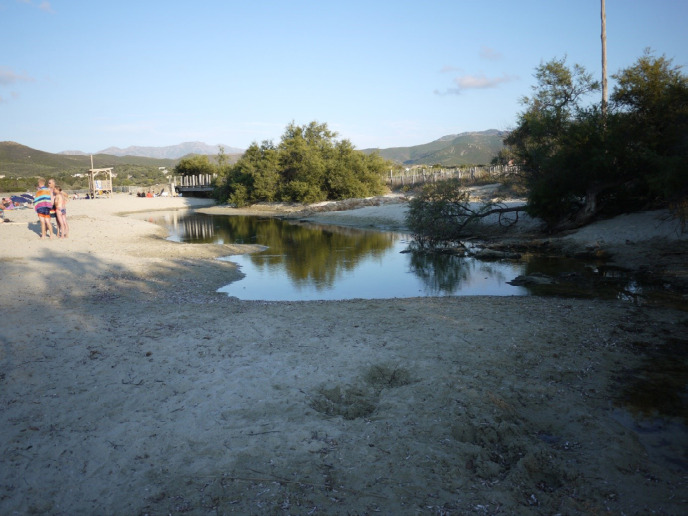
Embouchure de l'Osari fermée par un cordon lagunaire, côte occidentale, août 2022 (crédit photo: P Gazin) Osari river mouth closed by a sand barrier, West Coast, August 2022 (photo credit: P. Gazin)

**Figure 3:**
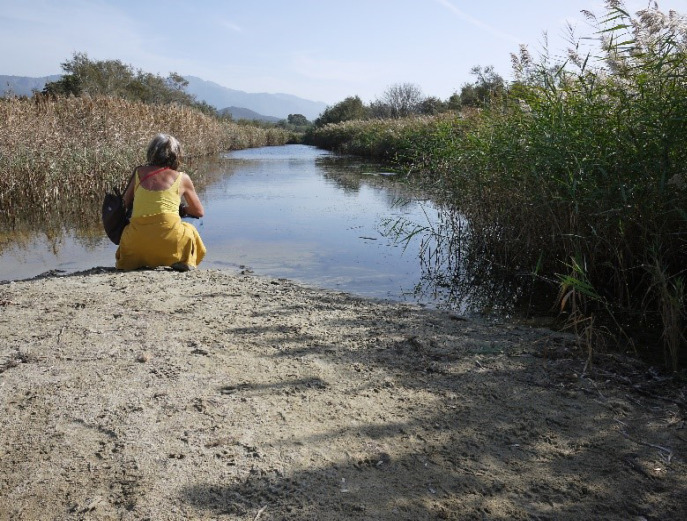
Embouchure de l'Ostriconi encore fermée par un cordon lagunaire début novembre 2022, une année déficitaire en pluie (crédit photo: P Gazin) Ostriconi river mouth sfili closed by a sand barrier at the beginning of November 2022, a year with unusually low rainfall (photo credit: P Gazin)

*An. labranchiae* du complexe *maculipennis* est confirmé comme principal vecteur, présent du littoral jusqu’à 500 m d'altitude, actif de mars à octobre, avec quelques adultes passant la saison froide dans l'habitat humain (Fig. 1). Canaux d'irrigation et de drainage mal entretenus, étangs, embouchures des fleuves côtiers, bassins d'arrosage, abreuvoirs constituent les gîtes larvaires (Fig. 2 et Fig. 3). Peu de déviation zoonotique, sauf parfois dans les cabanes à cochons.

**Figure 4:**
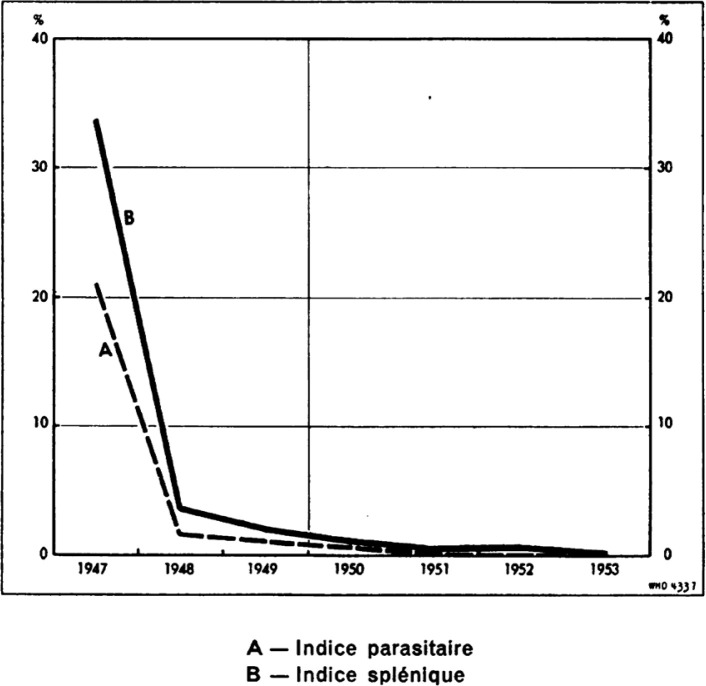
Évolution des indices plasmodique et splénique des moins de 16 ans de 1947 à 1953 [5] Evolution from 1947 to 1953 of the plasmodic and splenic indices of under 16 years of age *[5]*

Les activités contre les anophèles commencent en 1948 et reposent sur les aspersions intra-domiciliaires une fois par an principalement par du DDT (2 g/m^2^) dans toutes les pièces d'habitation, les rares étables, les porcheries et même les tombeaux ainsi que les tentes des campeurs. Le DDT est également utilisé en dispersion dans les gîtes aquatiques. Le faucardage des canaux et étangs se montre très efficace, les poissons *Gambusia* également. Le traitement par la quinine des cas fébriles ou suspects, notamment des sujets considérés comme plus fragiles (travailleurs immigrés, militaires) est largement pratiqué par trois dispensaires spécialisés ainsi que par la population elle-même en automédication. La décroissance des indices paludiques est très rapide (Fig. 4). En 1953, aucun nouveau cas de paludisme n'est officiellement enregistré.

Des cas autochtones sont cependant ultérieurement observés (20 cas en 1970, 10 en 1971, espèces non précisées) [2]. En 2006, un cas autochtone de *P. vivax* est décrit [3]. Depuis lors, aucune transmission autochtone de paludisme en Corse n'a été notifiée.

Ainsi, en 6 années de lutte antivectorielle intense couplée à une large utilisation de la quinine, la transmission des *Plasmodium* a été interrompue de manière durable en Corse. Des résultats équivalents ont été obtenus au cours des mêmes années en Sardaigne, en Grèce comme dans les Landes, en Charente, en Gironde et en Camargue… Cette durabilité a également largement bénéficié du remarquable développement des infrastructures, de l’éducation, de l'accès à la santé. Les armes employées ont été les mêmes que celles utilisées à partir des années 1950 en Afrique subsaharienne. Réussite d'un côté, échec de l'autre: l'intensité de l'anophélisme et la saisonnalité ont leur part de responsabilité, le développement économique et l’éducation, l'accès aux soins également.

La persistance d'un anophélisme important en Corse et l'introduction probable au cours de la saison chaude de *Plasmodium* par des porteurs de gamétocytes pourraient conduire à une reprise localisée de la transmission. Mais celle-ci n'a pas été constatée depuis 50 ans en dehors des rares exceptions citées. La rareté actuelle de porteurs de gamétocytes qui ne se traiteraient pas avec un antipaludique efficace peut être une des raisons de cet anophélisme persistant sans paludisme [11].

## Conclusion

La Corse a été, comme de nombreux autres territoires méditerranéens, une terre de paludisme endémique à *P. falciparum* et à *P. vivax*. Alphonse Laveran y a effectué à partir de 1899 plusieurs séjours qui lui ont confirmé la relation très forte entre la présence des anophèles et la présence de la maladie. Il y a rencontré des acteurs locaux décidés à débarrasser leur île de ce fléau et à participer à son développement. Les moyens utilisés, lutte contre les larves et les adultes, moustiquaires, traitements systématiques de la population ainsi que des malades, éducation à la santé sont toujours d'actualité 120 ans plus tard dans les zones où le paludisme sévit, désormais pour l'essentiel en Afrique subsaharienne. Ces actions et leurs résultats ont été fortement ralentis par les deux guerres mondiales. Des moyens financiers, une volonté politique, l'expérience acquise ont ensuite permis d'arrêter la transmission à partir de 1953. Aujourd'hui, la situation est celle d'un anophélisme sans transmission de *Plasmodium.* Il est possible que dans le futur des cas focalisés de paludisme apparaissent. La connaissance de la maladie par chacun en Corse et par ses professionnels de la santé permettrait une rapide contention de ces foyers.

## Liens D'intérêts

L'auteur ne déclare aucun lien d'intérêt.
